# Prediction Efficacy of Prognostic Nutritional Index and Albumin–Bilirubin Grade in Patients With Intrahepatic Cholangiocarcinoma After Radical Resection: A Multi-Institutional Analysis of 535 Patients

**DOI:** 10.3389/fonc.2021.769696

**Published:** 2021-12-10

**Authors:** Qi Li, Chen Chen, Jian Zhang, Hong Wu, Yinghe Qiu, Tianqiang Song, Xianhai Mao, Yu He, Zhangjun Cheng, Wenlong Zhai, Jingdong Li, Dong Zhang, Zhimin Geng, Zhaohui Tang

**Affiliations:** ^1^ Department of Hepatobiliary Surgery, The First Affiliated Hospital of Xi’an Jiaotong University, Xi’an, China; ^2^ Department of Hepatobiliary and Pancreatic Surgery, West China Hospital of Sichuan University, Chengdu, China; ^3^ Department of Biliary Surgery, Oriental Hepatobiliary Hospital Affiliated to Naval Medical University, Shanghai, China; ^4^ Department of Hepatobiliary Oncology, Tianjin Medical University Cancer Hospital, Tianjin, China; ^5^ Department of Hepatobiliary Surgery, Hunan Provincial People’s Hospital, Changsha, China; ^6^ Department of Hepatobiliary Surgery, The First Hospital Affiliated to Army Medical University, Chongqing, China; ^7^ Department of Hepatobiliary Surgery, Zhongda Hospital of Southeast University, Nanjing, China; ^8^ Hepatobiliary Pancreas and Liver Transplantation Surgery, The First Affiliated Hospital of Zhengzhou University, Zhengzhou, China; ^9^ Department of Hepatobiliary Surgery, Affiliated Hospital of North Sichuan Medical College, Nanchong, China; ^10^ Department of General Surgery, Xinhua Hospital Affiliated to Shanghai Jiaotong University School of Medicine, Shanghai, China

**Keywords:** intrahepatic cholangiocarcinoma, prognostic nutritional index, albumin–bilirubin grade, PNI+ALBI grade, nomogram

## Abstract

**Background:**

The preoperative nutritional status and the immunological status have been reported to be independent prognostic factors of patients with intrahepatic cholangiocarcinoma (ICC). This study aimed to investigate whether prognostic nutritional index (PNI) + albumin–bilirubin (ALBI) could be a better predictor than PNI and ALBI alone in patients with ICC after radical resection.

**Methods:**

The prognostic prediction evaluation of the PNI, ALBI, and the PNI+ALBI grade was performed in 373 patients with ICC who underwent radical resection between 2010 and 2018 at six Chinese tertiary hospitals, and external validation was conducted in 162 patients at four other Chinese tertiary hospitals. Overall survival (OS) and relapse-free survival (RFS) were estimated using the Kaplan–Meier method. Multivariate analysis was conducted to identify independent prognostic factors. A time-dependent receiver operating characteristic (ROC) curve and a nomogram prediction model were further constructed to assess the predictive ability of PNI, ALBI, and the PNI+ALBI grade. The C-index and a calibration plot were used to assess the performance of the nomogram models.

**Results:**

Univariate analysis showed that PNI, ALBI, and the PNI+ALBI grade were prognostic factors for the OS and RFS of patients with ICC after radical resection in the training and testing sets (*p* < 0.001). Multivariate analysis showed that the PNI+ALBI grade was an independent risk factor for OS and RFS in the training and testing sets (*p* < 0.001). Analysis of the relationship between the PNI+ALBI grade and clinicopathological characteristics showed that the PNI+ALBI grade correlated with obstructive jaundice, alpha-fetoprotein (AFP), cancer antigen 19-9 (CA19-9), cancer antigen 125 (CA125), PNI, ALBI, Child–Pugh grade, type of resection, tumor size, major vascular invasion, microvascular invasion, T stage, and N stage (*p* < 0.05). The time-dependent ROC curves showed that the PNI+ALBI grade had better prognostic predictive ability than the PNI, ALBI, and the Child–Pugh grade in the training and testing sets.

**Conclusion:**

Preoperative PNI+ALBI grade is an effective and practical predictor for the OS and RFS of patients with ICC after radical resection.

## Introduction

Intrahepatic cholangiocarcinoma (ICC) is the second most common biliary malignancy and accounts for about 10%–15% of primary liver carcinoma (1, 2). In recent years, the incidence of ICC has shown a significant upward trend worldwide (3, 4). At present, radical surgical resection represents the only potentially curative treatment option for ICC patients. However, survival remains poor even after curative hepatectomy due to tumor recurrence and metastasis, with the 5-year survival rate ranging from 20% to 40% (5, 6). Therefore, it is of great importance to screen new prognostic indicators to identify a high risk of recurrence or metastasis for ICC patients in order to provide clinical decision support.

The prognostic nutritional index (PNI), a widely used quantitative index for evaluating individual nutritional condition and inflammatory level, is calculated using the patient’s serum lymphocyte count and serum albumin level. It is normally used to evaluate the preoperative nutritional status of patients and assess individual surgical risk precisely. Albumin–bilirubin (ALBI) was first attempted to assess the liver function reserve of patients diagnosed with hepatocellular carcinoma (HCC) the same as the Child–Pugh grade in 2015, but ALBI had better predictive ability than the Child–Pugh grade for postoperative liver failure and long-term survival of patients undergoing liver resection (7, 8). Several recent studies have demonstrated that the PNI and ALBI are closely related to the prognosis of patients with HCC, ICC, and gallbladder cancer (8–13). Both the preoperative nutritional status and the immunological status have been reported to be independent prognostic factors of patients with ICC (11, 14–16). However, there is yet no conclusion on whether the combination of PNI and ALBI can improve the predictive ability of prognosis for ICC patients. This study aimed to investigate whether the PNI+ALBI grade could be a better predictor than PNI and ALBI alone in patients with ICC after radical resection.

## Material and Methods

### Patients

All patients who underwent curative resection and were pathologically confirmed to have ICC between 2010 and 2018 at 10 tertiary hospitals in China (West China Hospital of Sichuan University, Oriental Hepatobiliary Hospital Affiliated to Naval Medical University, Hunan Provincial People’s Hospital, The First Hospital Affiliated to Army Medical University, Zhongda Hospital of Southeast University, Xinhua Hospital Affiliated to Shanghai Jiaotong University School of Medicine, Tianjin Medical University Cancer Institute and Hospital, The First Affiliated Hospital of Xi’an Jiaotong University, The First Affiliated Hospital of Zhengzhou University, and Affiliated Hospital of North Sichuan Medical College) were considered for inclusion. The inclusion criteria were as follows: 1) patients ≥18 years old; 2) values for serum lymphocyte, albumin, and bilirubin were available; 3) patients underwent radical resection and the margin status recorded as microscopically negative (R0); 4) patients were without perioperative death. Every surgeon, with a title of professor or chief physician from high-volume medical centers in China, has undergone strict training and has proficient operation skills to avoid impacting the overall survival (OS) and relapse-free survival (RFS). All included patients were evaluated according to the 8th edition of the American Joint Committee on Cancer (AJCC) staging system and were followed up through December 2020.

The study was approved by the Ethics Committee of Xinhua Hospital Affiliated to Shanghai Jiaotong University School of Medicine (no. XHEC-JDYXY-2018-002), Shanghai, China, and the ethics committees of the other study centers. Written informed consent was obtained from all included patients and their families prior to study enrollment.

### Study Variables

The optimal cutoff values of OS and RFS for PNI and ALBI were calculated using the X-tile software (Yale University, New Haven, CT, USA). PNI and ALBI were calculated as follows: PNI = serum albumin (g/L) + 5 * total lymphocyte count (10^9^/L) (12, 17); ALBI = [log_10_bilirubin (μmol/L) * 0.66] + [albumin (g/L) * −0.085] (7). According to the results of X-tile, PNI ≤ 46.5 was defined as low PNI, while PNI > 46.5 was considered high; ALBI ≤ −2.70 was defined as low ALBI, while ALBI > −2.70 was considered high. With regard to the PNI+ALBI grade, a high PNI and a low ALBI were classified as grade A, a high PNI and a high ALBI or a low PNI and a low ALBI as grade B, and a low PNI and a high ALBI as grade C. A time-dependent receiver operating characteristic (time-ROC) analysis was conducted with R software version 3.6.1 (http://www.r-project.org/) to assess the prognostic predictive ability for OS and RFS of ICC patients. Other clinicopathological characteristics were also included in the survival analysis.

### Statistical Analysis

All statistical analyses were performed using SPSS version 25 (IBM Corp., Armonk, NY, USA). All continuous variables were expressed as the mean ± standard deviation. Categorical variables were examined using the *χ* ^2^ test. The Kaplan–Meier method and log-rank test were applied in the univariate analysis. The Cox proportional hazards regression model was used in the multivariate analysis. OS and RFS were calculated from the date of radical resection until the date of the most recent follow-up or death of the patient and as clinical evidence of tumor recurrence, respectively. Kaplan–Meier curves were calculated using GraphPad Prism (version 8.0; San Diego, CA, USA). A *p* < 0.05 was considered as statistically significant.

### Development and Assessment of Nomogram

All the included patients from 10 Chinese tertiary hospitals were split into a ratio of 7:3 to produce a training dataset from six hospitals (*N* = 373) and a testing dataset from the other four hospitals (*N* = 162). R software was used to produce nomogram prediction models for OS and RFS based on the same independent variables. The performance of the nomogram models was evaluated based on the concordance index (C-index) and a calibration plot.

## Results

A total of 535 curatively resected and pathologically confirmed ICC patients between 2010 and 2018 were considered for inclusion. Patients were aged from 22.0 to 83.0 years, with a median age of 59.0 years in the training set. Other characteristics of the cohort are shown on the left-hand side of **Table 1**. The 1-, 3-, and 5-year OS rates of patients were 80.7%, 41.5%, and 19.6%, and the 1-, 3-, and 5-year RFS rates were 51.5%, 17.9%, and 7.8%, respectively. The median OS and RFS were 19.10 and 12.00 months, respectively, in the training set.

**Table 1:** Univariate and multivariate analyses of the prognosis for intrahepatic cholangiocarcinoma (ICC) after radical resection.

	OS				RFS			
	Univariate analysis		Multivariate analysis		Univariate analysis		Multivariate analysis	
	HR (95%CI)	*p*-value	HR (95%CI)	*p*-value	HR (95%CI)	*p*-value	HR (95%CI)	*p*-value
Sex								
Female *vs*. Male	0.989 (0.755–1.295)	0.935			0.944 (0.751–1.187)	0.625		
Age (years)								
>55 *vs*. ≤55	1.447 (1.087–1.926)	0.011	1.521 (1.137–2.035)	0.005	1.041 (0.823–1.317)	0.737		
Obstructive jaundice								
Yes *vs*. no	1.027 (0.661–1.598)	0.905			0.912 (0.614–1.356)	0.651		
HBV infection								
Yes *vs*. no	0.792 (0.577–1.807)	0.148			1.045 (0.804–1.357)	0.744		
Hepatolithiasis								
Yes *vs*. no	1.844 (1.363–2.494)	<0.001	1.587 (1.160–2.171)	0.004	1.308 (1.000–1.710)	0.030	1.672 (1.225–2.282)	0.001
AFP (ng/ml)								
Abnormal *vs*. normal	1.364 (0.996–1.867)	0.053			1.086 (0.828–1.424)	0.552		
CEA (ng/ml)								
Abnormal *vs*. normal	1.402 (1.044–1.883)	0.025			1.297 (1.007–1.671)	0.044		
CA19-9 (U/ml)								
Abnormal *vs*. normal	1.373 (1.039–1.816)	0.026			1.308 (1.034–1.656)	0.025		
CA125 (U/ml)								
Abnormal *vs*. normal	1.329 (1.013–1.744)	0.040			1.554 (1.232–1.962)	<0.001		
PNI								
Low group *vs*. high group	1.900 (1.452–2.487)	<0.001			1.696 (1.340–2.147)	<0.001		
ALBI								
High group *vs*. low group	1.950 (1.488–2.556)	<0.001			1.776 (1.407–2.242)	<0.001		
PNI+ALBI grade								
Grade B *vs*. A	1.839 (1.290–2.621)	0.001	1.860 (1.295–2.672)	0.001	1.660 (1.232–2.237)	0.001	1.883 (1.312–2.703)	0.001
Grade C *vs*. A	2.238 (1.646–3.043)	<0.001	2.031 (1.476–2.796)	<0.001	1.921 (1.473–2.506)	<0.001	1.912 (1.396–2.619)	<0.001
Child–Pugh grade								
Grade B *vs*. A	1.273 (0.802–2.020)	0.305			1.167 (0.767–1.775)	0.470		
Type of resection								
Minor hepatectomy *vs*. wedge resection	1.460 (1.078–1.977)	0.014			1.434 (1.109–1.855)	0.006		
Major hepatectomy *vs*. wedge resection	1.854 (1.260–2.728)	0.002			1.763 (1.224–2.370)	0.002		
Lymphadenectomy								
Yes *vs*. no	1.061 (0.801–1.407)	0.680			1.140 (0.895–1.453)	0.289		
Tumor differentiation								
Moderate *vs*. well	2.007 (1.014–3.973)	0.045	1.651 (0.822–3.315)	0.038	1.587 (0.971–2.593)	0.045	1.578 (0.787–3.163)	0.045
Poor *vs*. well	3.294 (1.656–6.533)	0.001	2.985 (1.489–5.985)	0.002	2.001 (1.210–3.307)	0.007	2.847 (1.422–5.701)	0.003
Tumor location								
Right *vs*. left	0.886 (0.665–1.182)	0.411			1.054 (0.825–1.347)	0.674		
Left and right *vs*. left	0.852 (0.534–1.360)	0.502			1.038 (0.712–1.513)	0.847		
Morphologic grape								
Periductal infiltrating *vs*. mass-forming	0.869 (0.575–1.313)	0.505			0.788 (0.549–1.129)	0.194		
Intraductal growth *vs*. mass-forming	0.769 (0.453–1.251)	0.291			0.686 (0.463–1.015)	0.060		
Tumor size (cm)								
>5.0 *vs*. ≤5.0	1.534 (1.170–2.010)	0.002	1.475 (1.117–1.949)	0.006	1.363 (1.085–1.714)	0.008	1.469 (1.113–1.940)	0.007
Major vascular invasion								
Yes *vs*. no	1.821 (1.345–2.466)	<0.001			1.509 (1.151–1.978)	0.003		
Microvascular invasion								
Yes *vs*. no	1.783 (1.204–2.641)	0.004			1.566 (1.107–2.215)	0.011		
Perineural invasion								
Yes *vs*. no	2.125 (1.454–3.105)	<0.001	1.549 (1.035–2.319)	0.033	1.480 (1.063–2.060)	0.020	1.659 (1.111–2.476)	0.013
Liver capsule involvement								
Yes *vs*. no	1.264 (0.956–1.673)	0.021			1.571 (1.240–1.990)	<0.001		
Satellite nodules								
Yes *vs*. no	1.925 (1.345–2.754)	<0.001	2.068 (1.427–2.996)	<0.001	1.596 (1.167–2.181)	0.003	1.873 (1.305–2.689)	0.001
AJCC 8th edition T stage								
T_2_ *vs*. T_1a_/T_1b_	1.714 (1.199–2.449)	0.003			1.614 (1.202–2.168)	0.001		
T_3_/T_4_ *vs*. T_1a_/T_1b_	2.197 (1.505–3.205)	<0.001			1.943 (1.418–2.661)	<0.001		
AJCC 8th edition N stage								
N1 *vs*. N0	1.886 (1.402–2.536)	<0.001	1.609 (1.167–2.219)	0.004	1.554 (1.207–2.002)	0.001	1.691 (1.240–2.306)	0.001
AJCC 8th edition TNM stage								
II *vs*. IA/IB	1.573 (1.056–2.344)	0.026			1.446 (1.015–2.060)	0.041		
IIIA/IIIB/IV *vs*. IA/IB	1.836 (1.363–2.472)	<0.001			1.736 (1.354–2.227)	<0.001		
Adjuvant chemotherapy								
Yes *vs*. No	1.376 (1.044–1.814)	0.024			1.199 (0.945–1.521)	0.134		

OS, overall survival; RFS, recurrence-free survival; HR, hazard ratio; HBV, hepatitis B virus; AFP, alpha-fetoprotein; CEA, carcinoembryonic antigen; CA 19-9, cancer antigen 19-9; CA125, cancer antigen; PNI, prognostic nutritional index; ALBI, albumin–bilirubin; AJCC, American Joint Committee on Cancer.

### Survival Analysis for OS and RFS

**Figure 1:**
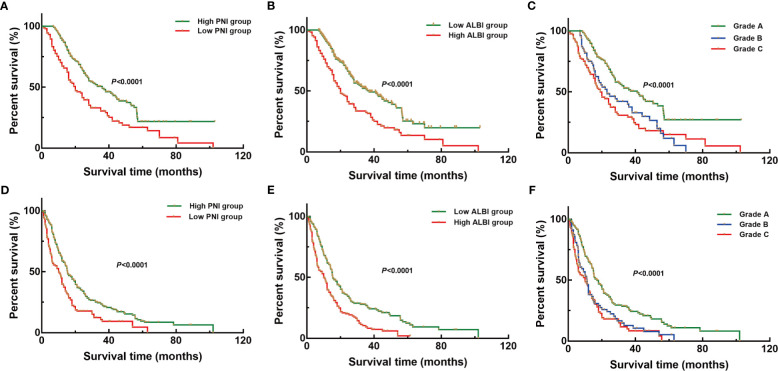
Kaplan–Meier overall survival (OS) and recurrence-free survival (RFS) curves of patients with intrahepatic cholangiocarcinoma (ICC) after radical resection according to different prognostic factors in the training set. **(A–C)** Kaplan–Meier OS curves according to the prognostic nutritional index (PNI) **(A)**, albumin–bilirubin (ALBI) **(B)**, and PNI+ALBI grade **(C)**. **(D–F)** Kaplan–Meier RFS curves according to the PNI **(D)**, ALBI **(E)**, and PNI+ALBI grade **(F)**.

The univariate analysis showed that PNI [OS: hazard ratio (HR) = 1.900, 95%CI = 1.452–2.487; RFS: HR = 1.696, 95%CI = 1.340–2.147], ALBI (OS: HR = 1.950, 95%CI = 1.488–2.556; RFS: HR = 1.776, 95%CI = 1.407–2.242), and the PNI+ALBI grade (OS: HR = 2.238, 95%CI = 1.646–3.043; RFS: HR = 1.921, 95%CI = 1.473–2.506) were prognostic factors for the OS and RFS of ICC patients after radical resection in the training set (*p* < 0.001; **Figure 1**). Multivariate analysis showed that PNI+ALBI grade (*HR*/95%CI = 2.031/1.476–2.796, *HR*/95%CI = 1.912/1.396–2.619) was independent risk factor for OS and RFS in training set (*p* < 0.001; **Figure 1**). Detailed results of the univariate and multivariate analyses are shown in **Table 1**.

**Figure 2:**
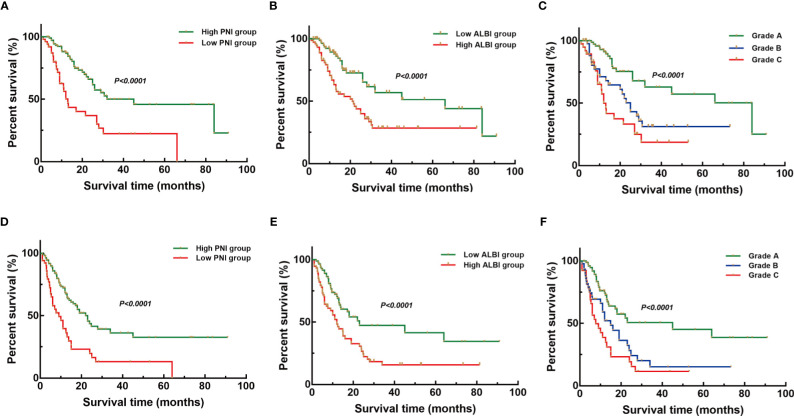
Kaplan–Meier overall survival (OS) and recurrence-free survival (RFS) curves of patients with intrahepatic cholangiocarcinoma (ICC) after radical resection according to different prognostic factors in the testing set. **(A–C)** Kaplan–Meier OS curves according to the prognostic nutritional index (PNI) **(A)**, albumin–bilirubin (ALBI) **(B)**, and PNI+ALBI grade **(C)**. **(D–F)** Kaplan–Meier RFS curves according to PNI **(D)**, ALBI **(E)**, and PNI+ALBI grade **(F)**.

Similarly, PNI, ALBI, and the PNI+ALBI grade were proven as prognostic factors and the PNI+ALBI grade as an independent risk factor for the OS and RFS of ICC patients after radical resection in the testing set (*p* < 0.001; **Figure 2**).

### Analysis of the Relationship Between PNI+ALBI Grade and Clinicopathological Characteristics

The PNI+ALBI grade correlated with obstructive jaundice, alpha-fetoprotein ((AFP), cancer antigen 19-9 (CA19-9), cancer antigen 125 (CA125), PNI, ALBI, Child–Pugh grade, type of resection, tumor size, major vascular invasion, microvascular invasion, and the 8th edition AJCC T stage and N stage (*p* < 0.05; **Table 2**).

**Table 2:** Relationship of PNI and ALBI with clinicopathological characteristics of intrahepatic cholangiocarcinoma (ICC) after radical resection.

	PNI (%)		*χ* ^2^	*p*-value	ALBI (%)		*χ* ^2^	*p*-value	PNI+ALBI grade (%)			*χ* ^2^	*p*-value
	High group	Low group			Low group	High group			Grade A	Grade B	Grade C		
Sex													
Male	108 (48.2)	72 (48.3)	0.000	0.984	83 (43.0)	97 (53.9)	4.418	0.036	72 (42.9)	47 (59.5)	61 (48.4)	5.958	0.051
Female	116 (51.8)	77 (51.7)			110 (57.0)	83 (46.1)			96 (57.1)	32 (40.5)	65 (51.6)		
Age (years)													
≤55	94 (42.0)	55 (36.9)	0.952	0.329	76 (39.4)	73 (40.6)	0.054	0.817	68 (40.5)	32 (40.5)	49 (38.9)	0.089	0.957
>55	130 (58.0)	94 (63.1)			117 (60.6)	107 (59.4)			100 (59.5)	47 (59.5)	77 (61.1)		
Obstructive jaundice													
No	212 (94.8)	126 (84.6)	10.691	0.001	187 (96.6)	151 (83.9)	18.518	<0.001	163 (97.0)	71 (89.9)	104 (82.5)	17.829	<0.001
Yes	12 (5.4)	23 (15.4)			6 (3.1)	29 (16.1)			5 (3.0)	8 (10.1)	22 (17.5)		
HBV infection													
No	164 (73.2)	115 (77.2)	0.747	0.387	143 (74.1)	136 (75.6)	0.106	0.745	121 (72.0)	63 (79.7)	95 (75.4)	1.736	0.420
Yes	60 (26.8)	34 (22.8)			50 (25.9)	44 (24.4)			47 (28.0)	16 (20.3)	31 (24.6)		
Hepatolithiasis													
No	179 (79.9)	104 (69.8)	4.998	0.025	153 (79.3)	130 (72.2)	2.530	0.112	135 (80.4)	60 (75.9)	88 (69.8)	4.350	0.114
Yes	45 (20.1)	45 (30.2)			40 (20.7)	50 (27.8)			33 (19.6)	19 (24.1)	38 (30.2)		
AFP (ng/ml)													
Normal	191 (25.3)	96 (64.4)	21.902	<0.001	163 (84.5)	124 (68.9)	12.722	<0.001	146 (86.9)	60 (75.9)	81 (64.3)	20.820	<0.001
Abnormal	33 (14.7)	53 (35.6)			30 (15.5)	56 (31.1)			22 (13.1)	19 (24.1)	45 (35.7)		
CEA (ng/ml)													
Normal	166 (74.1)	107 (71.8)	0.240	0.624	150 (77.7)	123 (68.3)	4.182	0.041	131 (78.0)	52 (65.8)	90 (71.4)	4.346	0.114
Abnormal	58 (25.9)	42 (28.2)			43 (22.3)	57 (31.7)			37 (22.0)	27 (34.2)	36 (28.6)		
CA19-9 (U/ml)													
Normal	99 (44.2)	47 (31.5)	6.014	0.014	89 (46.1)	57 (31.7)	8.161	0.004	82 (48.8)	24 (30.4)	40 (31.7)	12.031	0.002
Abnormal	125 (55.8)	102 (68.5)			104 (53.9)	123 (68.3)			86 (51.2)	55 (69.6)	86 (68.3)		
CA125 (U/ml)													
Normal	141 (62.9)	78 (52.3)	4.146	0.042	125 (64.8)	94 (52.2)	6.046	0.014	107 (63.7)	50 (63.3)	62 (49.2)	7.098	0.029
Abnormal	23 (37.1)	71 (47.7)			68 (35.2)	86 (47.8)			61 (36.3)	29 (36.7)	64 (50.8)		
PNI													
High group	–	–	–	–	169 (87.6)	55 (30.6)	126.181	<0.001	168 (100.0)	55 (69.6)	1 (0.8)	299.213	<0.001
Low group	–	–			24 (12.4)	125 (69.4)			0 (0)	24 (30.4)	125 (99.2)		
ALBI													
Low group	169 (75.4)	24 (16.1)	126.181	<0.001	–	–	–	–	168 (100.0)	24 (30.4)	1 (0.8)	302.110	<0.001
High group	55 (24.8)	125 (83.9)			–	–			0 (0)	55 (69.6)	125 (99.2)		
PNI+ALBI grade													
Grade A	168 (75.0)	0 (0)	299.213	<0.001	168 (87.0)	0 (0)	302.110	<0.001	–	–	–	–	–
Grade B	55 (24.6)	24 (16.1)			24 (12.4)	55 (30.6)			–	–	–		
Grade C	1 (0.4)	125 (83.9)			1 (0.5)	125 (69.4)			–	–	–		
Child–Pugh grade													
Grade A	217 (96.9)	129 (86.6)	14.132	<0.001	190 (98.4)	156 (86.7)	19.245	<0.001	166 (98.8)	73 (92.4)	107 (84.9)	20.703	<0.001
Grade B	7 (3.1)	20 (13.4)			3 (1.6)	24 (13.3)			2 (1.2)	6 (7.6)	19 (15.1)		
Type of resection													
Wedge resection	97 (43.3)	46 (30.9)	6.855	0.032	91 (47.2)	52 (28.9)	14.130	0.001	79 (47.0)	30 (38.0)	34 (27.0)	14.200	0.007
Minor hepatectomy	95 (42.4)	71 (47.7)			77 (39.9)	89 (49.4)			68 (40.5)	36 (45.6)	62 (49.2)		
Major hepatectomy	32 (14.3)	32 (21.5)			25 (13.0)	39 (21.7)			21 (12.5)	13 (16.5)	30 (23.8)		
Lymphadenectomy													
No	79 (35.3)	55 (36.9)	0.105	0.746	67 (34.7)	67 (37.2)	0.254	0.614	58 (34.5)	30 (38.0)	46 (36.5)	0.306	0.858
Yes	145 (64.7)	94 (63.1)			126 (65.3)	113 (62.8)			110 (65.5)	49 (62.0)	80 (63.5)		
Tumor differentiation													
Well	16 (7.1)	9 (6.0)	0.348	0.840	11 (5.7)	14 (7.8)	0.888	0.642	9 (5.4)	9 (11.4)	7 (5.6)	3.970	0.410
Moderate	125 (55.8)	81 (54.4)			110 (57.0)	96 (53.3)			97 (57.7)	41 (51.9)	68 (54.0)		
Poor	83 (37.1)	59 (36.9)			72 (37.3)	70 (38.9)			62 (36.9)	29 (36.7)	51 (40.5)		
Tumor location													
Left	113 (50.4)	77 (51.7)	0.354	0.838	98 (50.8)	92 (51.1)	0.181	0.913	86 (51.2)	37 (46.8)	67 (53.2)	2.194	0.700
Right	84 (37.5)	57 (38.3)			72 (37.3)	69 (38.3)			63 (37.5)	30 (38.0)	48 (38.1)		
Left and right	27 (12.1)	15 (10.1)			23 (11.9)	19 (10.6)			19 (11.3)	12 (15.2)	11 (8.7)		
Morphological grape													
Mass-forming	171 (76.3)	114 (76.5)	0.698	0.705	147 (76.2)	138 (76.7)	0.815	0.665	130 (77.4)	56 (70.9)	99 (78.6)	2.820	0.588
Periductal infiltrating	27 (12.1)	21 (14.1)			23 (11.9)	25 (13.9)			19 (11.3)	12 (15.2)	17 (13.5)		
Intraductal growth	26 (11.6)	14 (9.4)			23 (11.9)	17 (9.4)			19 (11.3)	11 (13.9)	10 (7.9)		
Tumor size (cm)													
≤5.0	127 (56.7)	73 (49.0)	2.135	0.144	112 (58.0)	88 (48.9)	3.130	0.077	95 (56.5)	49 (62.0)	56 (44.4)	7.089	0.029
>5.0	97 (43.3)	76 (51.0)			81 (42.0)	92 (51.1)			73 (43.5)	30 (38.0)	70 (55.6)		
Major vascular invasion													
No	186 (83.0)	111 (74.5)	4.022	0.045	159 (82.4)	138 (76.7)	1.876	0.171	139 (82.7)	67 (84.8)	91 (72.2)	6.569	0.037
Yes	38 (17.0)	38 (25.5)			34 (17.6)	42 (23.3)			29 (17.3)	12 (15.2)	35 (27.8)		
Microvascular invasion													
No	204 (91.1)	119 (79.9)	9.679	0.002	180 (93.3)	143 (79.4)	15.324	<0.001	157 (93.5)	68 (86.1)	98 (77.8)	15.263	<0.001
Yes	20 (8.9)	30 (20.1)			13 (6.7)	37 (20.6)			11 (6.5)	11 (13.9)	28 (22.2)		
Perineural invasion													
No	193 (86.2)	125 (83.9)	0.366	0.545	170 (88.1)	148 (82.2)	2.545	0.111	146 (86.9)	69 (87.3)	103 (81.7)	1.872	0.392
Yes	31 (13.8)	24 (16.1)			23 (11.9)	32 (17.8)			22 (13.1)	10 (12.7)	23 (18.3)		
Liver capsule involvement													
No	139 (62.1)	102 (68.5)	1.604	0.205	124 (64.2)	117 (65.0)	0.023	0.879	105 (62.5)	51 (64.6)	85 (67.5)	0.775	0.679
Yes	85 (37.9)	47 (31.5)			69 (35.8)	63 (35.0)			63 (37.5)	28 (35.4)	41 (32.5)		
Satellite nodules													
No	192 (85.7)	124 (83.5)	0.429	0.512	168 (87.0)	148 (82.2)	1.674	0.196	147 (87.5)	64 (81.0)	105 (83.3)	2.029	0.363
Yes	32 (14.3)	25 (16.8)			25 (13.0)	32 (17.8)			21 (12.5)	15 (19.0)	21 (16.7)		
AJCC 8th edition T stage													
T_1a_/T_1b_	64 (28.6)	33 (22.1)	4.659	0.097	59 (30.6)	38 (21.1)	6.211	0.045	49 (29.2)	25 (31.6)	23 (18.6)	9.543	0.049
T_2_	92 (41.1)	78 (52.3)			77 (39.9)	93 (51.7)			66 (39.3)	35 (44.3)	69 (54.8)		
T_3_/T_4_	68 (30.4)	38 (25.5)			57 (29.5)	49 (27.2)			53 (31.5)	19 (24.1)	34 (27.0)		
AJCC 8th edition N stage													
N0	171 (76.3)	103 (69.1)	2.387	0.122	154 (79.8)	120 (66.7)	8.230	0.004	134 (79.8)	55 (69.6)	85 (67.5)	6.346	0.042
N1	53 (23.7)	46 (30.9)			39 (20.2)	60 (33.3)			34 (20.2)	24 (30.4)	41 (32.5)		
AJCC 8th edition TNM stage													
IA/IB	99 (44.2)	62 (41.6)	4.340	0.114	92 (47.7)	69 (38.3)	3.315	0.191	78 (46.4)	35 (44.3)	48 (38.1)	4.402	0.354
II	25 (11.2)	28 (18.8)			25 (13.0)	28 (15.6)			19 (11.3)	10 (12.7)	24 (19.0)		
IIIA/IIIB/IV	100 (44.6)	59 (39.6)			76 (39.4)	83 (46.1)			71 (42.3)	34 (43.0)	54 (42.9)		

OS, overall survival; RFS, recurrence-free survival; HBV, hepatitis B virus; AFP, alpha-fetoprotein; CEA, carcinoembryonic antigen; CA 19-9, cancer antigen 19-9; CA125, cancer antigen; PNI, prognostic nutritional index; ALBI, albumin–bilirubin; AJCC, American Joint Committee on Cancer.

### Time-Dependent ROC Analysis

**Figure 3:**
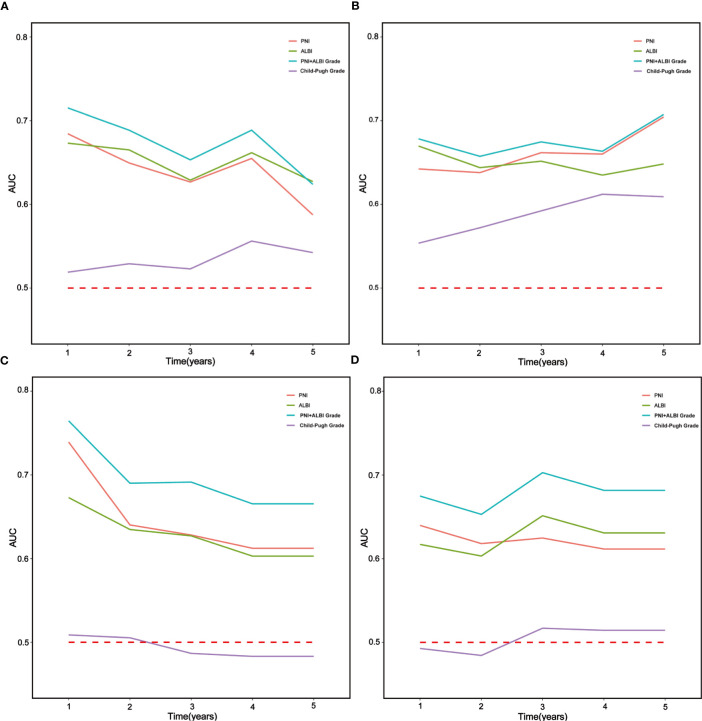
Time-dependent receiver operating characteristic (time-ROC) curves for overall survival (OS) and recurrence-free survival (RFS) of patients with intrahepatic cholangiocarcinoma (ICC) after radical resection. The *horizontal axis* shows the months after surgery and the *vertical axis* the estimated area under the ROC curve (AUC) for survival at the time of interest. *Blue*, *green*, *red*, and *purple solid lines* denote the estimated AUCs for the prognostic nutritional index (PNI) plus ALBI grade, ALBI, PNI, and Child–Pugh grade, respectively. **(A, C)** Time-ROC curves for OS in the training and testing sets, respectively. **(B, D)** Time-ROC curves for RFS in the training and testing sets, respectively.

The time-ROC curves are shown in **Figure 3**, which indicated that the PNI+ALBI grade had better prognostic predictive ability for OS and RFS than the PNI, ALBI, and the Child–Pugh grade in the training and testing sets.

### Development and Assessment of Nomogram

**Figure 4:**
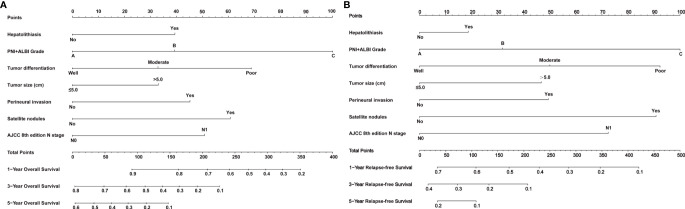
Nomogram prediction models for predicting the overall survival (OS) and recurrence-free survival (RFS) of patients with intrahepatic cholangiocarcinoma (ICC) after radical resection. **(A, B)** Nomograms for predicting the OS **(A)** and RFS **(B)** of patients with ICC after radical resection.

Nomogram prediction models for OS and RFS were established based on the same independent risk factors including the PNI+ALBI grade. Detailed results of the Cox regression are shown on the right-hand side of **Table 1**, and the nomogram models are shown in **Figure 4**. In addition, the online calculator of the nomogram models is shown in **Supplementary Figure 1** and available at https://doczj.shinyapps.io/onlinesur.

The C-index values of the nomogram models were 0.782 (95%CI = 0.730–0.834) and 0.773 (95%CI = 0.761–0.785) for OS in the training and testing sets, respectively. The C-index values were 0.736 (95%CI = 0.698–0.744) and 0.745 (95%CI = 0.733–0.757) for RFS in the training and testing sets, respectively. The calibration plots are shown in **Supplementary Figures 2**, **3**, which indicated that the nomogram models had better predictive ability in the training and testing sets.

## Discussion

The level of serum albumin, a well-recognized indicator, can reflect the nutritional status and liver function. It has many important physiological functions and regulates systemic inflammation (18). Lymphocytes, an enduring popular clinical indicator of the immune status of patients, play an important role in immune response to carcinoma and can mediate cytotoxic reaction and release cytokines to inhibit tumor growth, proliferation, and metastasis (19, 20). Therefore, PNI has an important theoretical basis in evaluating the prognosis of patients and is one of the important prognostic indicators for patients after surgery. In this study, a low PNI (≤46.5) tended to have worse OS and RFS than a high PNI (>46.5). Sayarlioglu et al. (21) have confirmed that there is a positive correlation between the patient’s serum albumin level and the total number of lymphocytes and the counts of CD4^+^ lymphocytes, which may be the reason for ICC patients with low PNI being prone to tumor recurrence and having poor prognosis after radical resection. Akgül et al. (11) and Zhang et al. (14) also confirmed that a low PNI was associated with a markedly worse prognosis for ICC patients. Recently, ALBI has drawn widespread attention in prognosis evaluation and has shown good prognostic predictive value. Similarly, a high ALBI (greater than −2.70) tended to have worse OS and RFS than a low ALBI (−2.70 or less) in this study. Several studies have also confirmed that a high ALBI has poor prognosis and is an independent risk factor for prognosis in patients with ICC (15, 16). Additionally, the time-ROC curves showed that ALBI was more accurate in predicting the OS and RFS of ICC patients than the Child–Pugh grade, which is consistent with the conclusion of Wang et al. (22) on advanced extrahepatic cholangiocarcinoma.

We further combined PNI with ALBI into the PNI-ALBI grade and classified it into grade A, grade B, and grade C, which can comprehensively assess the preoperative nutritional immunological status and liver function of patients with ICC. Pan et al. (23) first combined PNI with ALBI, but only classified it into a high PNI-ALBI grade and a low PNI-ALBI grade for early-stage HCC, which only showed the PNI-ALBI grade to have good predictive ability than the PNI or ALBI in the ROC curves. In this study, the PNI+ALBI grade was an independent risk factor for the OS and RFS of patients with ICC after radical resection, and PNI+ALBI grade C had worse OS and RFS than grades A and B. The time-ROC curves also showed that the PNI+ALBI grade had better prognostic predictive ability for OS and RFS than the PNI, ALBI, and the Child–Pugh grade in the training and testing sets. Therefore, the PNI+ALBI grade can be used as a more practical and reliable tool than the Child–Pugh grade for the prognostic evaluation of patients with ICC after radical resection.

By analyzing the relationship between PNI+ALBI grade and other clinicopathological characteristics, we concluded that the PNI+ALBI grade had a certain correlation with the clinicopathological characteristics related to both PNI and ALBI (such as obstructive jaundice, AFP, CA19-9, CA125, PNI, ALBI, Child–Pugh grade, type of resection, portal block time, and microvascular invasion) or related to PNI or ALBI only (such as blood loss, major vascular invasion, T stage, and N stage), and even had a certain correlation with characteristics not related to PNI or ALBI (such as tumor size). Therefore, the PNI+ALBI grade is associated with more clinicopathological characteristics than the PNI and ALBI separately. Moreover, the PNI+ALBI grade can not only effectively reflect the nutrition, inflammation levels, and liver function of patients but also assess the progress of ICC for prognostic evaluation.

To our knowledge, this study is the first to establish nomogram prediction models including the variable of PNI+ALBI grade. In these models, the PNI+ALBI grade was assigned the highest weighted score in the nomogram, which showed that the indicator had strong prognostic predictive ability compared to the other variables included in the models. In addition, the C-index values of the nomogram models were 0.782 and 0.736 for OS and RFS, respectively, which were superior to those of the nomogram models established by Wang et al. (24) and Hyder et al. (25).

However, there exist several limitations in our study. The sample size (535 patients included) was relatively modest; however this is consistent with the low incidence rate of ICC. In addition, it was difficult to avoid selection bias in the retrospective design because only patients who underwent radical resection were included. Accordingly, we recommend that future studies be conducted using larger samples and incorporating preoperative inflammatory biomarkers in order to explore preoperative nutrition, inflammation, and immunity-related biomarkers with stronger prognostic predictive ability. This, in turn, can provide the basis for clinical decision-making for ICC patients.

In conclusion, this study retrospectively analyzed 535 patients with ICC after radical resection and developed nomogram prediction models based on seven independent risk factors including the preoperative PNI+ALBI grade, which is an effective and practical predictor for OS and RFS in patients with ICC after radical resection. We expect that the preoperative PNI+ALBI grade will be increasingly effective for survival prediction for ICC in future studies and will eventually achieve widespread clinical application.

## Data Availability Statement

The raw data supporting the conclusions of this article will be made available by the authors, without undue reservation.

## Ethics Statement

The studies involving human participants were reviewed and approved by the Ethics Committee of Xinhua Hospital Affiliated to Shanghai Jiaotong University School of Medicine (no. XHEC-JDYXY-2018-002). The patients/participants provided written informed consent to participate in this study.

## Author Contributions

ZG and ZT conceived and designed the experiments and reviewed the manuscript. QL and CC performed the experiments. HW, YQ, TS, XM, YH, ZC, WZ, and JL collected and offered the data. QL and JZ contributed to analysis tools. QL, CC, JZ, and DZ conducted statistical analysis. QL and CC wrote the paper. All authors contributed to the article and approved the submitted version.

## Funding

This study was supported by the National Natural Science Foundation of China (no. 62076194 and no. 81772521), Multicenter Clinical Research Project of Shanghai Jiaotong University, School of Medicine (DLY201807), and the Clinical Training Program of Shanghai Xinhua Hospital Affiliated to Shanghai Jiaotong University, School of Medicine (17CSK06).

## Conflict of Interest

The authors declare that the research was conducted in the absence of any commercial or financial relationships that could be construed as a potential conflict of interest.

## Publisher’s Note

All claims expressed in this article are solely those of the authors and do not necessarily represent those of their affiliated organizations, or those of the publisher, the editors and the reviewers. Any product that may be evaluated in this article, or claim that may be made by its manufacturer, is not guaranteed or endorsed by the publisher.

## Supplementary Material

Supplementary Figure 1 The online calculator of the nomogram models for patients with ICC after radical resection based on PNI+ALBI grade.

Supplementary Figure 2 The calibration plots for predicting 1-, 3- and 5-year OS and RFS of patients with ICC after radical resection in training set. Calibration plot of nomogram prediction of **(A)** 1-year, **(B)** 3-year and **(C)** 5-year OS, and **(D)** 1-year, **(E)** 3-year and **(F)** 5-year RFS of patients with ICC after radical resection.

Supplementary Figure 3 The calibration plots for predicting 1-, 3- and 5-year OS and RFS of patients with ICC after radical resection in testing set. Calibration plot of nomogram prediction of **(A)** 1-year, **(B)** 3-year and **(C)** 5-year OS, and **(D)** 1-year, **(E)** 3-year and **(F)** 5-year RFS of patients with ICC after radical resection.
