# Supplementary figures and images for: Prediction Efficacy of Prognostic Nutritional Index and Albumin–Bilirubin Grade in Patients With Intrahepatic Cholangiocarcinoma After Radical Resection: A Multi-Institutional Analysis of 535 Patients
Source: Front Oncol. 2021 Dec 10;11:769696. doi: 10.3389/fonc.2021.769696 (PMC8702533; doi:10.3389/fonc.2021.769696)

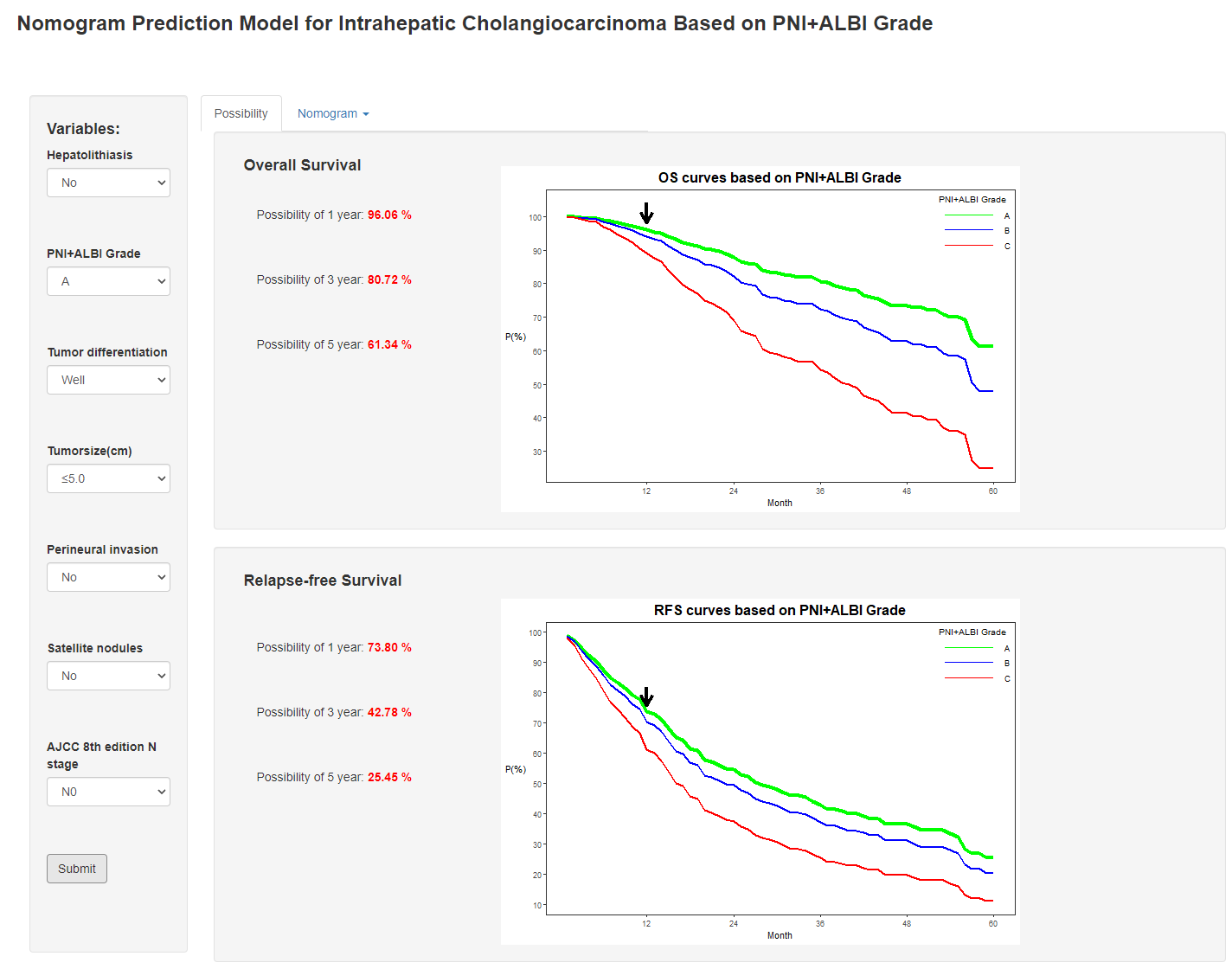

Supplement: Supplementary Figure 1 — The online calculator of the nomogram models for patients with ICC after radical resection based on PNI+ALBI grade. [file Image_1.tif]

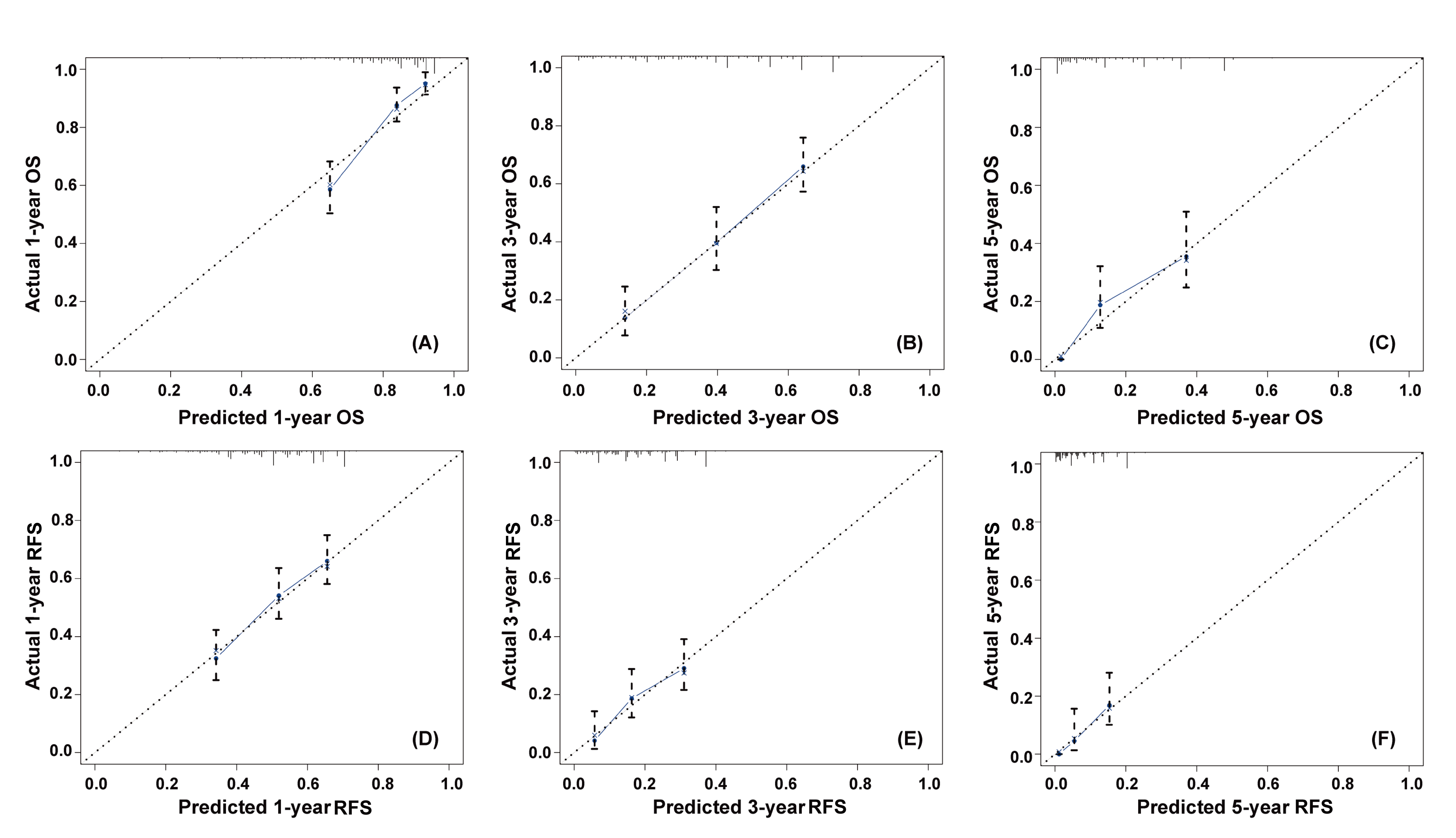

Supplement: Supplementary Figure 2 — The calibration plots for predicting 1-, 3- and 5-year OS and RFS of patients with ICC after radical resection in training set. Calibration plot of nomogram prediction of (A) 1-year, (B) 3-year and (C) 5-year OS, and (D) 1-year, (E) 3-year and (F) 5-year RFS of patients with ICC after radical resection. [file Image_2.tif]

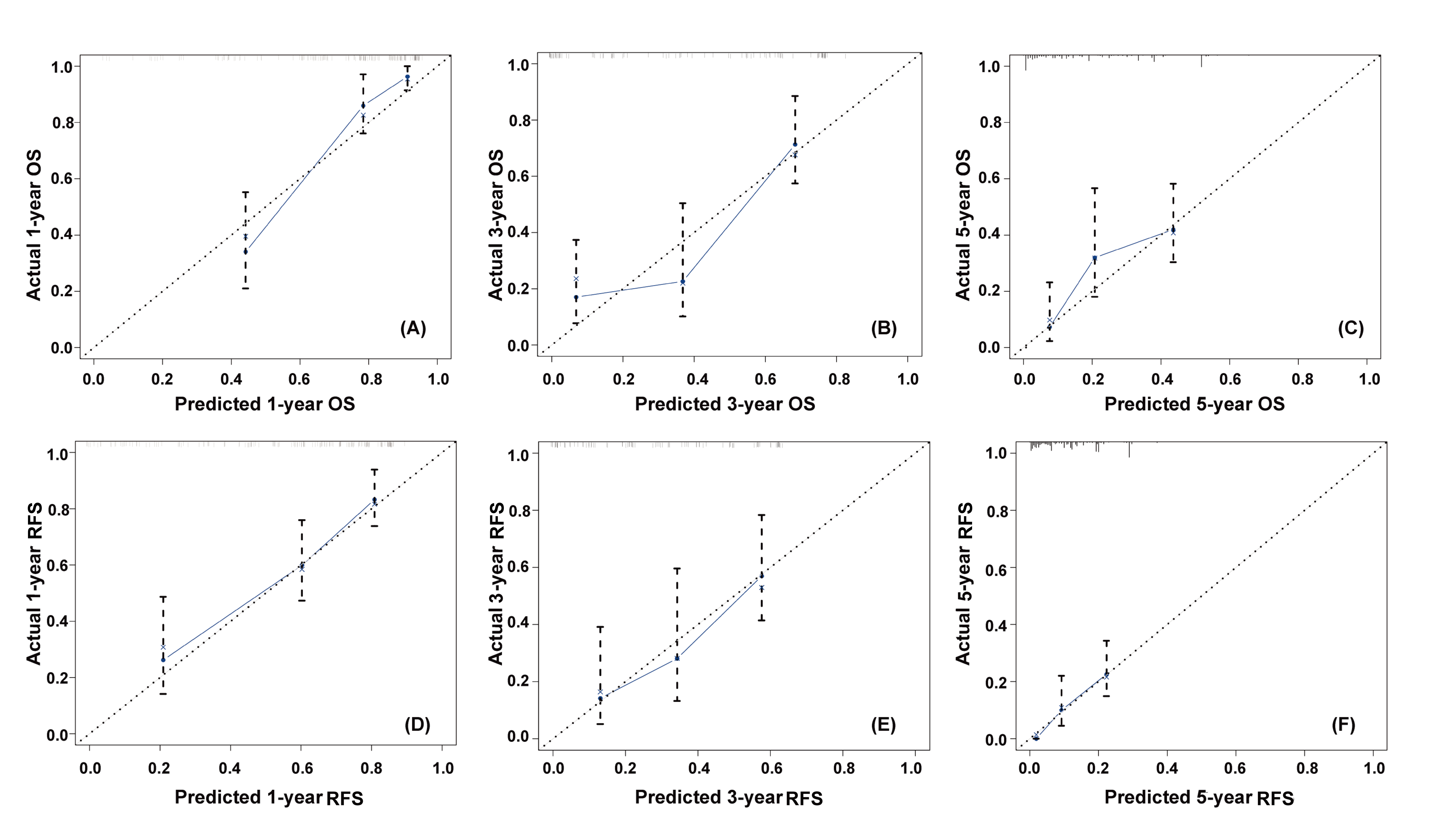

Supplement: Supplementary Figure 3 — The calibration plots for predicting 1-, 3- and 5-year OS and RFS of patients with ICC after radical resection in testing set. Calibration plot of nomogram prediction of (A) 1-year, (B) 3-year and (C) 5-year OS, and (D) 1-year, (E) 3-year and (F) 5-year RFS of patients with ICC after radical resection. [file Image_3.tif]
