# Supplementary material for: Prediction Efficacy of Prognostic Nutritional Index and Albumin–Bilirubin Grade in Patients With Intrahepatic Cholangiocarcinoma After Radical Resection: A Multi-Institutional Analysis of 535 Patients
Source: Front Oncol. 2021 Dec 10;11:769696. doi: 10.3389/fonc.2021.769696 (PMC8702533; doi:10.3389/fonc.2021.769696)
Supplement: Supplementary file 4 [file Table_1.docx]

Supplement table Comparison of Training Set and Testing Set for ICC after Radical Resection

|  | **Training set (%)** |  | **Testing set (%)** |  | **χ^2^** |  | **P value** |
| --- | --- | --- | --- | --- | --- | --- | --- |
| Sex |  |  |  |  |  |  |  |
| Male | 180(48.3) |  | 90(55.6) |  | 2.407 |  | 0.121 |
| Female | 193(51.7) |  | 72(44.4) |  |  |  |  |
| Age (year) |  |  |  |  |  |  |  |
| ≤55 | 149(39.9) |  | 72(44.4) |  | 0.943 |  | 0.332 |
| >55 | 224(60.1) |  | 90(55.6) |  |  |  |  |
| Obstructive jaundice |  |  |  |  |  |  |  |
| No | 338(90.6) |  | 150(92.6) |  | 0.550 |  | 0.458 |
| Yes | 35(9.4) |  | 12(7.4) |  |  |  |  |
| HBV infection |  |  |  |  |  |  |  |
| No | 279(74.8) |  | 121(74.7) |  | 0.001 |  | 0.979 |
| Yes | 94(25.2) |  | 41(25.3) |  |  |  |  |
| Hepatolithiasis |  |  |  |  |  |  |  |
| No | 283(75.9) |  | 135(83.3) |  | 3.681 |  | 0.055 |
| Yes | 90(24.1) |  | 27(16.7) |  |  |  |  |
| AFP (ng/ml) |  |  |  |  |  |  |  |
| ≤7.0 | 287(76.9) |  | 127(78.4) |  | 0.136 |  | 0.712 |
| >7.0 | 86(23.1) |  | 35(21.6) |  |  |  |  |
| CEA (ng/ml) |  |  |  |  |  |  |  |
| ≤5.0 | 273(73.2) |  | 117(72.2) |  | 0.054 |  | 0.817 |
| >5.0 | 100(26.8) |  | 45(27.8) |  |  |  |  |
| CA19-9(U/ml) |  |  |  |  |  |  |  |
| ≤39.0 | 146(36.1) |  | 72(44.4) |  | 1.315 |  | 0.251 |
| >39.0 | 227(60.9) |  | 90(55.6) |  |  |  |  |
| CA125(U/ml) |  |  |  |  |  |  |  |
| ≤35.0 | 219(58.7) |  | 94(58.0) |  | 0.022 |  | 0.882 |
| >35.0 | 154(41.3) |  | 68(42.0) |  |  |  |  |
| PNI |  |  |  |  |  |  |  |
| High group | 224(60.1) |  | 110(67.9) |  | 2.966 |  | 0.085 |
| Low group | 149(39.9) |  | 52(32.1) |  |  |  |  |
| ALBI |  |  |  |  |  |  |  |
| Low group | 193(51.7) |  | 87(53.7) |  | 0.174 |  | 0.676 |
| High group | 180(48.3) |  | 75(46.3) |  |  |  |  |
| PNI+ALBI Grade |  |  |  |  |  |  |  |
| Grade A | 168(45.0) |  | 78(48.1) |  | 5.809 |  | 0.055 |
| Grade B | 79(21.2) |  | 45(27.8) |  |  |  |  |
| Grade C | 126(33.8) |  | 39(24.1) |  |  |  |  |
| Child-Pugh Grade |  |  |  |  |  |  |  |
| Grade A | 346(92.8) |  | 153(94.4) |  | 0.510 |  | 0.475 |
| Grade B | 27(7.2) |  | 9(5.6) |  |  |  |  |
| Type of resection |  |  |  |  |  |  |  |
| Wedge resection | 143(38.3) |  | 71(43.8) |  | 3.030 |  | 0.220 |
| Minor hepatectomy | 166(44.5) |  | 59(36.4) |  |  |  |  |
| Major hepatectomy | 64(17.2) |  | 32(19.8) |  |  |  |  |
| Lymphadenectomy |  |  |  |  |  |  |  |
| No | 134(35.9) |  | 70(43.2) |  | 2.541 |  | 0.111 |
| Yes | 239(64.1) |  | 92(56.8) |  |  |  |  |
| Tumor differentiation |  |  |  |  |  |  |  |
| Well | 25(6.7) |  | 12(7.4) |  | 4.068 |  | 0.131 |
| Moderate | 206(55.2) |  | 103(63.6) |  |  |  |  |
| Poor | 142(38.1) |  | 47(29.0) |  |  |  |  |
| Tumor location |  |  |  |  |  |  |  |
| Left | 180(50.9) |  | 73(45.1) |  | 1.569 |  | 0.456 |
| Right | 141(37.8) |  | 69(42.6) |  |  |  |  |
| Left and Right | 42(11.3) |  | 20(12.3) |  |  |  |  |
| **Morphologic grape** |  |  |  |  |  |  |  |
| Mass-forming | 285(76.4) |  | 139(85.8) |  | 7.374 |  | 0.025 |
| Periductal infiltrating | 48(12.9) |  | 16(9.9) |  |  |  |  |
| Intraductal growth | 40(10.7) |  | 7(4.3) |  |  |  |  |
| Tumor size (cm) |  |  |  |  |  |  |  |
| ≤5.0 | 175(46.9) |  | 71(43.8) |  | 0.434 |  | 0.510 |
| >5.0 | 198(53.1) |  | 91(56.2) |  |  |  |  |
| Major vascular invasion |  |  |  |  |  |  |  |
| No | 297(79.6) |  | 140(86.4) |  | 3.485 |  | 0.062 |
| Yes | 76(20.4) |  | 22(13.6) |  |  |  |  |
| Microvascular invasion |  |  |  |  |  |  |  |
| No | 323(86.6) |  | 136(84.0) |  | 0.648 |  | 0.421 |
| Yes | 50(13.4) |  | 26(16.0) |  |  |  |  |
| Perineural invasion |  |  |  |  |  |  |  |
| No | 318(85.3) |  | 140(86.4) |  | 0.124 |  | 0.724 |
| Yes | 55(14.7) |  | 22(13.6) |  |  |  |  |
| Liver capsule involvement |  |  |  |  |  |  |  |
| No | 241(64.6) |  | 112(69.1) |  | 1.030 |  | 0.310 |
| Yes | 132(35.4) |  | 50(30.9) |  |  |  |  |
| Satellite nodules |  |  |  |  |  |  |  |
| No | 316(84.7) |  | 133(82.1) |  | 0.575 |  | 0.448 |
| Yes | 57(15.3) |  | 29(17.9) |  |  |  |  |
| AJCC 8th edition T stage |  |  |  |  |  |  |  |
| T_1a_/T_1b_ | 97(26.0) |  | 39(24.1) |  | 4.594 |  | 0.101 |
| T_2_ | 170(45.6) |  | 89(54.9) |  |  |  |  |
| T_3_/T_4_ | 106(28.4) |  | 34(21.0) |  |  |  |  |
| AJCC 8th edition N stage |  |  |  |  |  |  |  |
| N0 | 274(73.5) |  | 109(67.3) |  | 2.117 |  | 0.146 |
| N1 | 99(26.5) |  | 53(32.7) |  |  |  |  |
| AJCC 8th edition TNM stage |  |  |  |  |  |  |  |
| IA/IB | 161(43.2) |  | 70(43.2) |  | 1.871 |  | 0.392 |
| II | 53(14.2) |  | 30(18.5) |  |  |  |  |
| IIIA/IIIB/IV | 159(42.6) |  | 62(38.3) |  |  |  |  |
| **Adjuvant chemotherapy** |  |  |  |  |  |  |  |
| No | 251(67.3) |  | 56(34.6) |  | 49.459 |  | <0.001 |
| Yes | 122(32.7) |  | 106(65.4) |  |  |  |  |
